# Predictive and Prognostic Brain Metastases Assessment in Luminal Breast Cancer Patients: FN14 and GRP94 from Diagnosis to Prophylaxis

**DOI:** 10.3389/fonc.2017.00283

**Published:** 2017-12-01

**Authors:** Antonio Martínez-Aranda, Vanessa Hernández, Ferran Moreno, Núria Baixeras, Daniel Cuadras, Ander Urruticoechea, Miguel Gil-Gil, Noemí Vidal, Xavier Andreu, Miquel A. Seguí, Rosa Ballester, Eva Castella, Angels Sierra

**Affiliations:** ^1^Biological Clues of the Invasive and Metastatic Phenotype Group, Bellvitge Biomedical Research Institute – IDIBELL, L’Hospitalet de Llobregat, Barcelona, Spain; ^2^Departament de Medicina, Hospital Universitari Vall d’Hebron, Universitat Autònoma de Barcelona, Barcelona, Spain; ^3^Servei d’Oncologia Radioteràpica, Institut Català d’Oncologia (ICO), Hospital Duran i Reynals, L’Hospitalet de Llobregat, Barcelona, Spain; ^4^Servei d’Anatomia Patològica, Hospital Universitari de Bellvitge, L’Hospitalet de Llobregat, Barcelona, Spain; ^5^Statistical Service, Sant Joan de Déu Research Foundation, Barcelona, Spain; ^6^Breast Cancer Unit, Institut Català d’Oncologia – IDIBELL, Hospital Duran i Reynals, L’Hospitalet de Llobregat, Barcelona, Spain; ^7^Neuroncology Unit, Institut Català d’Oncologia – IDIBELL, Hospital Duran i Reynals, L’Hospitalet de Llobregat, Barcelona, Spain; ^8^Servei d’Anatomia Patològica, Consorci Hospitalari Parc Taulí, Barcelona, Spain; ^9^Servei d’Oncología Mèdica, Consorci Hospitalari Parc Taulí, Barcelona, Spain; ^10^Servei d’Oncología Radioteràpica, Institut Català d’Oncologia (ICO), Hospital Universitari Germans Trias i Pujol, Barcelona, Spain; ^11^Servei d’Anatomia Patològica de Can Ruti, Institut Català d’Oncologia (ICO), Hospital Universitari Germans Trias i Pujol, Barcelona, Spain; ^12^Laboratory of Molecular and Translational Oncology, Institut d’Investigacions Biomèdiques August Pi i Sunyer-IDIBAPS, Centre de Recerca Biomèdica CELLEX, Barcelona, Spain; ^13^Faculty of Medicine, Universitat de VIC-Universitat Central de Catalunya, Barcelona, Spain

**Keywords:** biomarkers, brain metastasis, breast cancer, FN14, GRP94, prediction, prevention, prognosis

## Abstract

FN14 has been implicated in many intracellular signaling pathways, and GRP94 is a well-known endoplasmic reticulum protein regulated by glucose. Recently, both have been associated with metastasis progression in breast cancer patients. We studied the usefulness of FN14 and GRP94 expression to stratify breast cancer patients according their risk of brain metastasis (BrM) progression. We analyzed FN14 and GRP94 by immunohistochemistry in a retrospective multicenter study using tissue microarrays from 208 patients with breast carcinomas, of whom 52 had developed BrM. Clinical and pathological characteristics and biomarkers expression in *Luminal* and *non-Luminal* patients were analyzed using a multivariate logistic regression model adjusted for covariates, and brain metastasis-free survival (BrMFS) was estimated using the Kaplan–Meier method and the Cox proportional hazards model. FN14 expression was associated with BrM progression mainly in *Luminal* breast cancer patients with a sensitivity (53.85%) and specificity (89.60%) similar to Her2 expression (46.15 and 89.84%, respectively). Moreover, the likelihood to develop BrM in FN14-positive *Luminal* carcinomas increased 36.70-fold (3.65–368.25, *p* = 0.002). Furthermore, the worst prognostic factor for BrMFS in patients with *Luminal* carcinomas was FN14 overexpression (HR = 8.25; 95% CI: 2.77–24.61; *p* = 0.00015). In these patients, GRP94 overexpression also increased the risk of BrM (HR = 3.58; 95% CI: 0.98–13.11; *p* = 0.054—Wald test). Therefore, FN14 expression in *Luminal* breast carcinomas is a predictive/prognostic biomarker of BrM, which combined with GRP94 predicts BrM progression in *non-Luminal* tumors 4.04-fold (1.19–8.22, *p* = 0.025), suggesting that both biomarkers are useful to stratify BrM risk at early diagnosis. We propose a new follow-up protocol for the early prevention of clinical BrM of breast cancer patients with BrM risk.

## Introduction

Identification of molecular subtypes has enhanced our understanding of breast cancer biology (1), overcoming one of the main barriers to improving the progression, prognosis, and treatment of breast cancer, namely, its clinical and genetic heterogeneity. The gene expression patterns derived from cDNA microarrays of primary breast carcinomas have made it possible to correlate tumor characteristics with clinical outcome (2) and support the idea that breast tumor subtypes represent biologically distinct disease entities with different survival rates (3). The main recognized breast cancer subtypes are as follows: *Luminal A*, estrogen-receptor (ER) positive, Ki-67 < 14%, and normal expression of Her2; *Luminal B*, ER-positive, Ki-67 ≥ 14%, and normal expression of Her2; *Luminal/Her2*+, ER-positive and Her2 overexpression; *Her2-enriched*, ER-negative and Her2 overexpression; and *triple negative (TN)*, ER-negative, progesterone receptor (PR) negative, and normal expression of Her2. One of the important differences between subtypes as regards clinical progression is that hormone receptor-positive tumors, such as *Luminal A*, have a better prognosis for survival compared with Her2 overexpression and *TN* subtypes (4, 5) and the lowest risk of lymph node metastasis, whereas the *Luminal-Her2*+ subtype has the highest risk (6). Moreover, hormone receptor-positive subtypes such as *Luminal A* and *Luminal B* should be considered different oncologic entities sharing similarities when studying their pattern of response to therapy (7). Breast cancer molecular subtypes are used to stratify patients at increased risk of recurrence, who may benefit from more aggressive local treatment (8–10). For example, the *Luminal/Her2*+ and *Her2-enriched* subtypes are associated with a significantly higher rate of brain, lung, and liver metastases in comparison with the *Luminal A* subtype, whereas *TN* patients are associated with a higher rate of brain, lung and distant nodal metastases (11–14).

Despite improvements in diagnosis and novel adjuvant therapies, brain metastasis (BrM) is becoming a serious clinical problem, with a higher incidence in patients with histological grade (HG) 3, high Ki-67 expression (15), age younger than 50 years old (11, 16), ER-negative and Her2-positive (11, 17). Breast cancer subtypes also determine the prognosis and survival of a patient with BrM (18, 19). Patients with *Luminal* tumors have a better survival rate than those with *TN* tumors (20), whereas those with the *Her2-enriched* subtype have a significantly poorer prognosis than those with *Luminal/Her2*+ or *Luminal* tumors (21). Patients with *TN* tumors have worse overall and disease-free survival rates (22), especially in patients with lung metastases. Even patients with non-metastatic *TN* breast cancer have a high early risk of developing BrM as a first site of recurrence (23), and worse survival after brain radiotherapy (24) than those with the *non-TN* phenotype. In these patients, BrM represents a significant adverse prognostic factor not only to overall survival but also to neurologic and radiosurgical survival (25).

We recently reported BrM biomarkers that discriminate breast carcinomas according to their likelihood of BrM progression, regardless of whether or not they expressed Her2 (26, 27). Of these, GRP94 (94 kDa glucose-regulated protein), a signaling regulator and a major endoplasmic reticulum chaperone and FN14 (fibroblast growth factor-inducible protein) implicated in many intracellular signaling pathways, both have been implicated in the promotion of tumor proliferation and metastasis.

GRP94 has calcium binding properties that are conferring its major function in protein folding, assembly and degradation (28, 29). Tumor hypoxia activates endoplasmic reticulum stress upregulating the unfolded protein response (30). The expression of GRP94 correlates with advanced stage and poor survival in many cancers (31, 32).

In addition, FN14 is implicated in several signaling pathways that control the cancer hallmarks (33). Typically, reactive astrocytes produce proinflammatory cytokines, among them TWEAK (TNF-like weak inducer of apoptosis), a type II membrane protein which activates FN14 (34). The binding of TWEAK to FN14 is involved in regulating perivascular astrocytes and the blood–brain barrier interface (35). Moreover, FN14 has been involved in cachexia and the treatment with anti-FN14 antibodies improves body and muscle mass and adipose tissue in mice, increasing survival and general welfare (36).

Given these results, we hypothesized that the expression of FN14 and GRP94 could be used for early identification of the risk of breast cancer brain metastasis, whatever the molecular subtype. Thus, we studied their expression in breast cancer primary tumors according to their molecular subtype defined by Her2, ER, PR, and Ki-67 expression. Our results indicate that FN14 is the most useful predictive/prognostic biomarker of BrM in breast cancer patients with *Luminal* (*Luminal A, Luminal B*, and *Luminal/Her2*+) carcinomas. Moreover, in combination with GRP94, FN14 predicts also BrM progression in *non-Luminal* tumors.

## Materials and Methods

### Patients

We obtained 211 samples from patients diagnosed between 1989 and 2009 (Table S1 in Supplementary Material) at the following institutions: Catalan Institute of Oncology (I.C.O.), Hospital Duran i Reynals and the Hospital Universitari de Bellvitge (L’Hospitalet de Llobregat, Spain); Consorci Hospitalari Parc Taulí (Sabadell, Spain); and I.C.O., Hospital Universitari Germans Trias i Pujol (Badalona, Spain). A final total of 208 patients were included in this study, whose clinical (Table S2 in Supplementary Material), pathological parameters and molecular subtype status were as follows (Table S3 in Supplementary Material): 40.3% *Luminal A* (ER-positive, Ki-67 < 14%); 18.5% *Luminal B* (ER-positive, Ki-67 ≥ 14%); 9.0% *Luminal/Her2*+ (ER-positive and Her2 overexpressed), 10.0% *Her2-enriched* (ER-negative and Her2 overexpressed), and 22.2% *TN* tumors (ER-negative, PR-negative, and normal expression of Her2). Some missing values from these variables discarded three patients, two from *Luminal* tumors and one from *non-Luminal* tumors.

For this work involving databases of human information we followed the Spanish National law on the protection of Personal Data “Ley 15/1999, 13 Dec.”

### Histology and Immunohistochemistry

Tissue microarrays (TMAs) were prepared from three representative areas of the tumor, as described previously (27). Primary antibodies anti-GRP94 at 1/2,000 and anti-FN14 at 1/3,000 (Santa Cruz Biotechnology, Santa Cruz, CA, USA) were diluted in Dako Real™ Antibody Diluent Buffer (Dakocytomation, Dako, Denmark): Tris buffer, pH 7.2, 15 mM Na_3_N. LSAB + System-HRP (Dakocytomation) was used for staining, including secondary antibodies in PBS, streptavidin conjugated to HRP in PBS, and liquid 3,3′ diaminobenzidine in chromogen solution.

**Figure 1:**
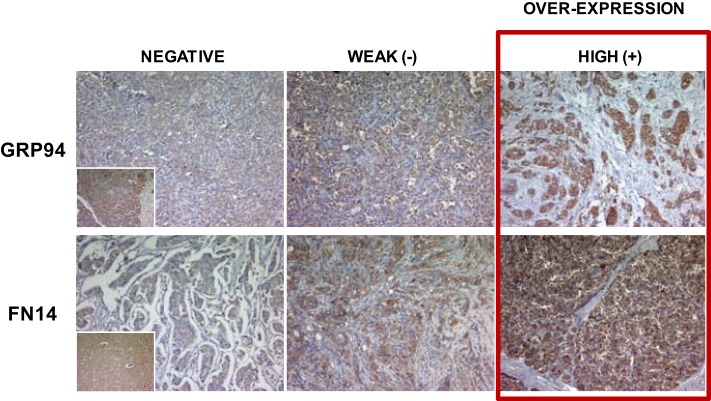
Representative tabulation of protein expression in breast cancer samples. Representative staining of GRP94 and FN14 and tabulation of protein expression in breast cancer samples. Tissues are shown as viewed by light microscopy. Negative and weak intensities of staining were considered both negative for semi-quantitative purposes to avoid false positive samples, and only tumors with unequivocal high intensity staining were considered as positive overexpression. Original magnification 10×. *Insets*: standard positive control tissue sample used in each determination.

The overexpression of GRP94 and FN14 was categorized as positive when strong expression was detected and negative when no or weak expression was detected, to avoid false positives (Figure 1). Morphologic diagnosis was performed with classical hematoxylin–eosin staining.

### Statistics

Frequencies of categorical variables were compared among groups using the χ^2^-test or Fisher’s exact test where appropriate. Brain metastasis-free survival (BrMFS) was estimated for each group using the Kaplan–Meier method and was compared among them using the Cox proportional hazards model, estimating their hazard ratio and 95% CI.

To evaluate the correlation between BrM and protein expression, immunostained samples were graded on a three-category scale as follows: negative, weak positive, and strong positive. The marker was classified as overexpressed only in strong positive samples to avoid false positives. Biomarker sensitivity and specificity, both singly and in combination, was assessed in both *Luminal* and *non-Luminal* patients. The biomarkers combinations were considered positive when at least one of them was positive and negative when all of them were negative.

A multivariate logistic regression analysis adjusted for covariates was carried out in both *Luminal* and *non-Luminal* groups to study in patients with BrM vs. NBrM the presence of biomarkers (GRP94 and FN14) in their primary tumor. The covariates used were as follows: age (≥50, 40–49, and <40), positive axillary nodes (0, 1–3, and ≥4), Her2 status (negative and positive), and presence of lung metastasis (no and yes), where the first category mentioned for each variable was the reference. We calculated the OR associated with the biomarker, its 95% CI and *p*-value.

In this analysis, the variable “triple negative (no, yes)” was not included as a covariate because all patients belonging to the *Luminal* group were “no” for this variable. Moreover, Her2 status was not included as a covariate when the combination (GRP94 + FN14 + Her2) was used as the biomarker.

Values were considered significant when *p* was less than 0.05. Software used: R Core Team (37).

## Results

### Clinical Characteristics of Breast Carcinoma Subtypes and BrM Involvement

We studied patient characteristics according to three different groups of progression patterns (Table S1 in Supplementary Material): *brain metastases* (BrM), with or without metastases (WoM) at other sites; *non-brain distant metastases* (NBrM), patients with metastasis in bones and/or liver and/or lungs and/or non-regional lymph nodes, but not in brain; and patients WoM. The distribution of breast cancer molecular subtypes changed across the three groups of patients. The main distinctive parameters that characterized the BrM group were *age*, whereby below 50 years old was significantly different (*p* = 0.001); *hormone receptor negativity* [both ER (*p* < 0.0001) and PR (*p* < 0.0001)], an attribute of tumors that developed BrM in contrast to tumors from NBrM and WoM patients; and *Her2 positivity* and a *high Ki-67 index* (*p* = 0.01 and *p* < 0.0001, respectively). Other parameters, such as *tumor size* (*p* = 0.001), *HG* (*p* < 0.0001), and *lymph node involvement* (*p* < 0.0001) were similar among BrM and NBrM patients, but different in WoM patients.

More than 50% of BrM patients had *TN* tumors. By contrast, only 20.6% of NBrM and 9.8% of WoM patients had *TN* tumors (χ^2^-test: *p* < 0.0001). Important differences were observed in the *Her2-enriched* subtype with regard to metastasis progression, occurring in 23.1% of BrM, 8.8% of NBrM, and 4.9% of WoM groups. Moreover, the distribution of *Luminal/Her2*+ tumors was different in patients according metastasis involvement, being 11.5% in BrM, 8.8% in NBrM, and 8.2% in WoM groups. Therefore, according to the clinical and pathological characteristics of breast cancer patients, we established two main groups due to limited size of our sample: the *Luminal group* (*Luminal A, Luminal B*, and *Luminal/Her2*+ subtypes), which encompassed 25% (13/52) of patients with BrM, 70.6% (24/34) with NBrM and 85.3% (104/122) of WoM patients; and the *non-Luminal* group (*TN* and *Her2-enriched* subtypes), encompassing 75% (39/52) of patients with BrM, 29.4% (10/34) with NBrM, and 14.7% (18/122) of WoM patients. The clinical and pathological parameters of the *Luminal* and *non-Luminal* groups are described in Supplementary Table S4 in Supplementary Material: *tumor size*, ≥21 mm in 32.61 vs. 55.56%, respectively (*p* = 0.002); *HG* 3, 32.12 and 81.25%, respectively (*p* < 0.0001); and *regional lymph node* involvement (affected ≥4), in 14.39 vs. 35.39%, respectively (*p* = 0.0008).

As expected, significant differences were related to the therapeutic approach adopted: 81.82% of *Luminal* patients vs. 58.21% of *non-Luminal* patients did not receive neoadjuvant chemotherapy (*p* = 0.0002); 83.22 vs. 55.88% (*p* < 0.0001) received conservative surgery; 44.76 vs. 18.03% (*p* = 0.001) did not receive adjuvant chemotherapy; and 85.92 vs. 6.06% (*p* < 0.0001) received tamoxifen or another antiestrogen as hormonal therapy, respectively. Moreover, clinical relapses, with the presence of local (8.39 vs. 19.12%, *p* = 0.024), regional (1.40 vs. 16.18%, *p* < 0.0001), or distant relapses (27.27 vs. 73.53%, *p* < 0.0001) were less frequent in patients from the *Luminal* group than in those in the *non-Luminal* group, respectively.

The incidence of BrM (Table 1) was significantly higher in the *non-Luminal* group than in the *Luminal* group [58.21% (39/67) vs. 9.22% (13/141), *p* < 0.0001]. Furthermore, patient outcomes corroborated the clinical and pathological characteristics, with an increase in metastasis-free survival when tumors were *Luminal* in both BrM and NBrWoM groups (Figures S1A,B in Supplementary Material). Moreover, the worst prognosis was found in patients with *non-Luminal* tumors (HR = 10.57, 95% CI: 5.60–19.96; and HR = 4.01, 95% CI: 2.52–6.38; respectively, *p* < 0.0001). These results indicate that the assessment of subtypes in our series provided an effective subclassification according to BrM progression in patients, similar to other reported series (11, 14).

**Table 1:** Distribution of patients with brain metastasis, other metastases (NBrM), and non-metastasis (without metastases) according to molecular subtype of the primary breast tumor.

Patients (*N* = 208)^a^				

Characteristics	Brain metastases	Non-brain distant metastases	Without metastases	*p*-Value (χ^2^-test)

	*N* (%)	*N* (%)	*N* (%)	

	52 (25.0)	34 (16.3)	122 (58.7)	
**Molecular subtypes**				
Triple negative	27 (51.9)	7 (20.6)	12 (9.8)	
Her2-enriched	12 (23.1)	3 (8.8)	6 (4.9)	
Lum/Her2+	6 (11.5)	3 (8.8)	10 (8.2)	
Luminal B	5 (9.6)	8 (23.6)	25 (20.5)	
Luminal A	2 (3.9)	13 (38.2)	69 (56.6)	<0.0001

*^a^Patients included in these three categories (*N* = 208/211). Three patients were missing (not suitable for biomarkers assessment)*.

### FN14 and GRP94 Stratify Breast Cancer Molecular Subtypes According to Their BrM Progression Risk

Since molecular subtypes in NBrM (tumors with bone, liver, lung, skin, etc.) and WoM had a similar clinic-pathological parameters distribution (Fisher’s exact test, *p* = 0.230; data not shown), we considered both as a single group (NBrWoM) to better compare the expression of BrM biomarkers between *Luminal* (*N* = 141) and *non-Luminal* (*N* = 67) tumors (see Table 2). FN14 was overexpressed in 14.5% (20/138) of *Luminal* tumors and in 23.1% (15/65) of *non-Luminal* tumors. Moreover, in *Luminal* tumors, FN14 expression was significantly different between the BrM and NBrWoM groups, at 53.8% (7/13) vs. 10.4% (13/125), respectively (Fisher’s exact test, *p* = 0.0005). FN14 expression in *non-Luminal* tumors differed between BrM and NBrWoM patients, being 31.6% (12/38) vs. 11.1% (3/27), respectively, although not significantly (Fisher’s exact test, *p* = 0.07). Therefore, we concluded that FN14 expression in *Luminal* breast tumors was associated with BrM progression. GRP94 was overexpressed in 47.5% (66/139) of *Luminal* and 50% (33/66) of *non-Luminal* tumors. Biomarkers expression in *Luminal* tumors was also significantly different between BrM and NBrWoM patients, being 76.9% (10/13) vs. 44.4% (56/126) (Fisher’s exact test; *p* = 0.04), whereas GRP94 expression in the *non-Luminal* group was similar for BrM, at 57.9% (22/38) of patients, and NBrWoM at 39.3% (11/28) (Fisher’s exact test; *p* = 0.21). These results highlight the intrinsic value of FN14 and GRP94 as organ-specific BrM biomarkers mainly in patients with tumors from the *Luminal* subtype.

**Table 2:** Distribution of biomarkers in brain metastasis (BrM) and NBrWoM patients in both *Luminal* and *non-Luminal* groups.

Patients (*N* = 208)				

Biomarker	Luminal group (*N* = 141)		Non-Luminal group (*N* = 67)	
	BrM	NBrWoM^a^	BrM	NBrWoM^a^
	
	*N* (%)	*N* (%)	*N* (%)	*N* (%)
FN14+^b^	7/13 (53.8)	13/125 (10.4)	12/38 (31.6)	3/27 (11.1)
	Fisher’s exact test (*p* = 0.0005)		Fisher’s exact test (*p* = 0.07)	

GRP94+^c^	10/13 (76.9)	56/126 (44.4)	22/38 (57.9)	11/28 (39.3)
	Fisher’s exact test (*p* = 0.04)		Fisher’s exact test (*p* = 0.21)	

*^a^This category encompasses those patients “without metastases” and those with “non-brain distant metastases” [bone, lung, liver, and non-regional lymph node metastases; in this category, five patients were previously excluded because they only had skin metastasis (*n* = 2), pleural metastasis (*n* = 2), or meningeal metastasis (*n* = 1)]*.

*^b^FN14 biomarker was assessed in 138 patients belonging to the *Luminal* group (this was not possible in 3 of them) and in 65 from the *non-Luminal* group (this was not possible in 2 of them)*.

*^c^GRP94 biomarker was assessed in 139 patients belonging to the *Luminal* group (this was not possible in 2 of them) and in 66 from the *non-Luminal* one (this was not possible for 1 of them)*.

*Differences shown in number of patients (*N*) in each group are due to patients whose biomarkers were not available*.

Furthermore, we analyzed in tumors with BrM progression if the expression of BrM biomarkers were associated with Her2 overexpression. We found that FN14 expression was independent of Her2 status in patients with BrM from the *Luminal* group (Fisher’s exact test; *p* = 0.59). By contrast, FN14 expression in the *non-Luminal* group was associated in 63.6% of cases with the *Her2-enriched* subtype (Table S5 in Supplementary Material), differing from *TN* tumors, of which 18.5% were positive for FN14 (Fisher’s exact test, *p* = 0.017).

GRP94 expression in tumors from BrM affected patients was independent of Her2 status (Table S5 in Supplementary Material) in the *Luminal* (Fisher’s exact test; *p* = 1.00) and *non-Luminal* groups (Fisher’s exact test, *p* = 0.30).

### FN14 and GRP94 Are Prognostic Biomarkers for BrM in Luminal Tumors

First, we studied metastasis-free survival in our series according to molecular subtype (Figure S2 in Supplementary Material), using the Kaplan–Meier method and the Cox proportional hazards model. The *TN* subtype was used as a reference group for comparative purposes. We analyzed the distribution of subtypes in NBrM patients (Figure S2A in Supplementary Material). Differences between the *TN* and *Her2-enriched* subtypes were not statistically significant (HR = 0.81; 95% CI: 0.41–1.60; *p* = 0.53). By contrast, *Luminal/Her2*+ (HR = 0.41; 95% CI: 0.18–0.96; *p* = 0.039), *Luminal B* (HR = 0.33; 95% CI: 0.17–0.64; *p* = 0.0012), and *Luminal A* (HR = 0.16; 95% CI: 0.08–0.30; *p* < 0.0001) showed a significantly lower incidence of NBrM than *TN* breast carcinomas. Furthermore, in BrM patients, we found significant differences in BrMFS with regard to molecular subtype, using the *TN* subtype as the reference group (Figure S2B in Supplementary Material): *Her2-enriched* did not differ significantly from *TN* (HR = 0.79; 95% CI: 0.40–1.55; *p* = 0.49), but the remaining subtypes showed a significantly lower risk than *TN*: *Luminal/Her2*+: HR = 0.33; 95% CI: 0.13–0.80; *p* = 0.014. *Luminal B*: HR = 0.13; 95% CI: 0.05–0.33; *p* < 0.0001 and *Luminal A*: HR = 0.02; 95% CI: 0.005–0.09; *p* < 0.0001.

**Figure 2:**
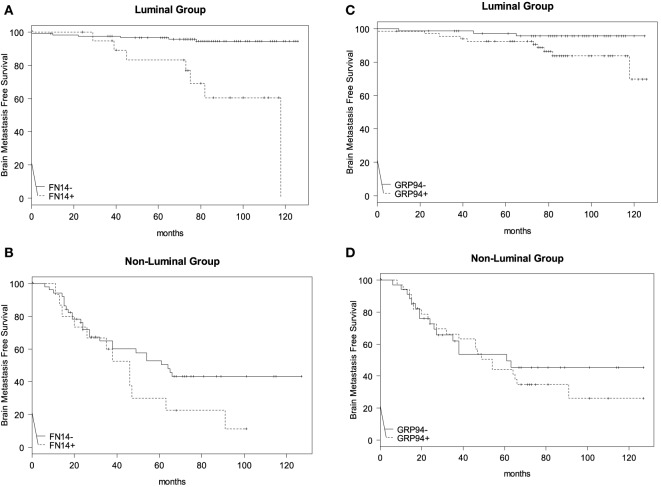
Brain metastasis-free survival (BrMFS) in breast cancer patients according to expression of FN14 and GRP94 biomarkers. Comparisons between *Luminal* **(A)** and *non-Luminal* **(B)** groups, according to FN14 positivity in primary tumors revealed a shortened BrMFS that was statistically significant in the *Luminal* group (HR = 8.25; 95% CI: 2.77–24.61; *p* = 0.00015) but not in the *non-Luminal* one (HR = 1.74; 95% CI: 0.87–3.47; *p* = 0.11). With regard to GRP94 positivity, shortened BrMFS in *Luminal* **(C)** and *non-Luminal* **(D)** groups was not statistically significant (HR = 3.58; 95% CI: 0.98–13.11; *p* = 0.054 and HR = 1.22; 95% CI: 0.64–2.32; *p* = 0.55, respectively).

Next, we studied BrMFS in the *Luminal* group according to whether the tumor expressed FN14 or not (Figure 2A), and we found that overexpression of FN14 was associated with a reduction in BrMFS (HR = 8.25; 95% CI: 2.77–24.61; *p* = 0.00015). Although, different stratification of the *non-Luminal* group (Figure 2B) according to FN14 expression was not statistically significant (HR = 1.74; 95% CI: 0.87–3.47; *p* = 0.11).

In addition, we analyzed BrMFS in the *Luminal* group according to GRP94 expression (Figure 2C) and observed that GRP94 was overexpressed in tumors from patients with reduced BrMFS (HR = 3.58; 95% CI: 0.98–13.11; *p* = 0.054—Wald test), although this finding did not reach statistical significance either. Meanwhile, BrMFS according to GRP94 expression in *non-Luminal* tumors was not significantly different (HR = 1.22; 95% CI: 0.64–2.32; *p* = 0.55) (Figure 2D).

### FN14 Is a Predictive Biomarker of BrM in Luminal Tumors

Since overexpression of the Her2 gene is associated with a higher risk to develop BrM, we analyzed the sensitivity and specificity of FN14 and GRP94 expression to predict BrM and compared these parameters to the prediction given by Her2 in these patients (Table 3). In patients belonging to the *Luminal* group (low risk of BrM *a priori*), FN14 and Her2 showed more specificity (89.60 and 89.84%, respectively) than GRP94 (55.56%) to predict BrM progression. However, GRP94 expression showed more sensitivity (76.92%) than FN14 and Her2 (53.85 and 46.15%, respectively) to predict BrM involvement.

**Table 3:** Sensitivity and specificity of biomarkers, singly or in combination, with regard to brain metastases in *Luminal* and *non-Luminal* patients.

Patients (*N* = 208)						

Biomarker	Luminal group (*N* = 141)			Non-Luminal group (*N* = 67)		
	*N*^b^	Sensitivity (%)	Specificity (%)	*N*^b^	Sensitivity (%)	Specificity (%)
FN14	138	(53.85)	(89.60)	65	(31.58)	(88.89)
GRP94	139	(76.92)	(55.56)	66	(57.89)	(60.71)
Her2	141	(46.15)	(89.84)	67	(30.77)	(67.86)
FN14 + GRP94^a^	139	(84.62)	(50.00)	66	(68.42)	(57.14)
FN14 + GRP94 + Her2^a^	139	(84.62)	(45.24)	67	(71.79)	(42.86)

*^a^In each case, these combinations were considered positive when at least one of the assessed molecules was positive and negative when all of them were negative*.

*^b^Differences in number or patients (*N*) are due to those patients whose biomarker assessment (singly or in combination) was unknown*.

On the other hand, in patients with a higher risk of BrM, such as *non-Luminal* ones, FN14 (88.89%) was the most specific protein to discriminate tumors that developed BrM, followed by Her2 (67.86%) and GRP94 (60.71%). GRP94 sensitivity was again higher (57.89%) than that obtained with FN14 or Her2 (31.58 and 30.77%, respectively).

Expression of the biomarker combination FN14 + GRP94 improved BrM risk assessment in *Luminal* patients compared with *non-Luminal* ones (sensitivity: 84.62 and 68.42%, specificity: 50 and 57.14%, respectively). The addition of Her2 yielded 84.62 and 71.79% sensitivity, and 45.24 and 42.86% specificity in *Luminal* with regard to *non-Luminal*, respectively (Table 3). The combination of FN14 + GRP94 + Her2 increased sensitivity to predict BrM in both *Luminal* and *non-Luminal* cases, covering a wide range of patients.

The high specificity shown by FN14 and Her2 when evaluated singly was lost in combination, suggesting that both biomarkers stratified different patient subgroups of BrM risk.

To assess the usefulness of FN14 and GRP94 as independent risk factors of BrM, a multivariate analysis was performed, including age, axillary node involvement, Her2 status, and presence of lung metastasis as covariates in the analysis (Table 4).

**Table 4:** Multivariate analysis of biomarkers in patients with brain metastasis from the *Luminal* and *non-Luminal* groups.

	Patients (*N* = 199)^a^			
Biomarker	Luminal group (*N* = 136)		Non-Luminal group (*N* = 63)	
	OR (95% CI)	*p*	OR	(95% CI)	*p*
FN14	36.70 (3.65–368.25)	0.002	3.29 (0.64–16.79)	0.153
GRP94	5.74 (0.87–37.66)	0.069	3.23 (0.98–10.63)	0.053
FN14 + GRP94^b^	7.10 (0.88–57.51)	0.066	4.04 (1.19–13.65)	0.025
FN14 + GRP94 + Her2^b^	5.80 (0.86–39.08)	0.071	2.45 (0.73–8.22)	0.147

*The covariates used in this study were as follows: age, axillary lymph node involvement, Her2 status, and presence of lung metastasis. Her2 was not included as a covariate when we used the combination GRP94 + FN14 + Her2 as biomarker*.

*^a^The number of patients (*N*) obtained in each group corresponds to those patients who presented all the required covariates*.

*^b^In each case, these combinations were considered positive when at least one of the assessed molecules was positive and negative when all of them were negative*.

Outcomes showed that patients belonging to the *Luminal* group (ER+) had a statistically significant higher risk to develop BrM if the tumor expressed FN14 compared with those with FN14 negative tumors (OR: 36.70; 95% CI: 3.65–368.25; *p* = 0.002). The likelihood of these patients to develop BrM increased 36.70-fold. By contrast, when tumors overexpressed GRP94, the risk to develop BrM (OR = 5.74; 95% CI: 0.87–37.66) remained non-significant (*p* = 0.069), either in combination with FN14 (*p* = 0.066) or Her2 (*p* = 0.071) positivity (OR = 7.10, 95% CI: 0.88–57.51; and OR = 5.80, 95% CI: 0.86–39.08, respectively).

In *non-Luminal* patients, only the combination of FN14 and GRP94 positivity was significantly predictive of BrM progression (95% CI: 1.19–13.65, *p* = 0.025), with a 4.04-fold likelihood to develop BrM (Table 4). These results are in consonance with our previous results reporting the usefulness of both biomarkers to predict BrM in *TN* breast cancer patients (27).

In summary, our study reveals that (1) A subset of breast cancer patients with a better prognosis *a priori*, such as estrogen-receptor positive with or without Her2 positivity, develop brain metastases if FN14 and/or GRP94 biomarkers are positive in their primary tumor. (2) FN14-positive status impairs the prognosis of breast cancer patients by shortening the length of BrMFS. (3) The likelihood to develop BrM in patients with FN14-positive tumors increases 36.70-fold (*p* = 0.002). (4) The combined assessment of biomarkers (FN14 + GRP94) shows a higher benefit in risk evaluation of BrM in patients with *non-Luminal* tumors.

Since stratification impairs statistical consistency, further multicentre studies with wider amount of patients are needed to reinforce the results.

## Discussion

The routine analysis of ER, PR, Ki-67, and Her2 status in breast tumors can predict relapse, providing the standard approach for clinical decision-making in the adjuvant setting (17–19). However, these procedures are insufficient to predict BrM.

This study provides evidence that a subset of breast cancer patients with a better prognosis *a priori*, such as patients with *Luminal* carcinomas, with or without Her2 positivity, can be stratified by their likelihood to develop BrM if FN14 is overexpressed (OR = 36.70). Thus, the clinical use of FN14 expression might facilitate a preventive strategy for patients at high risk for BrM progression and will improve the design of trials aimed at its prevention. Moreover, the combined assessment of both FN14 and GRP94 proteins shows a higher benefit in risk evaluation of BrM progression, especially in *non-Luminal* patients (4.04-fold), independently of Her2 status (OR = 2.45). Therefore, the use of FN14 and GRP94 expression at early diagnosis might stratify those BrM patients prone to BrM.

**Figure 3:**
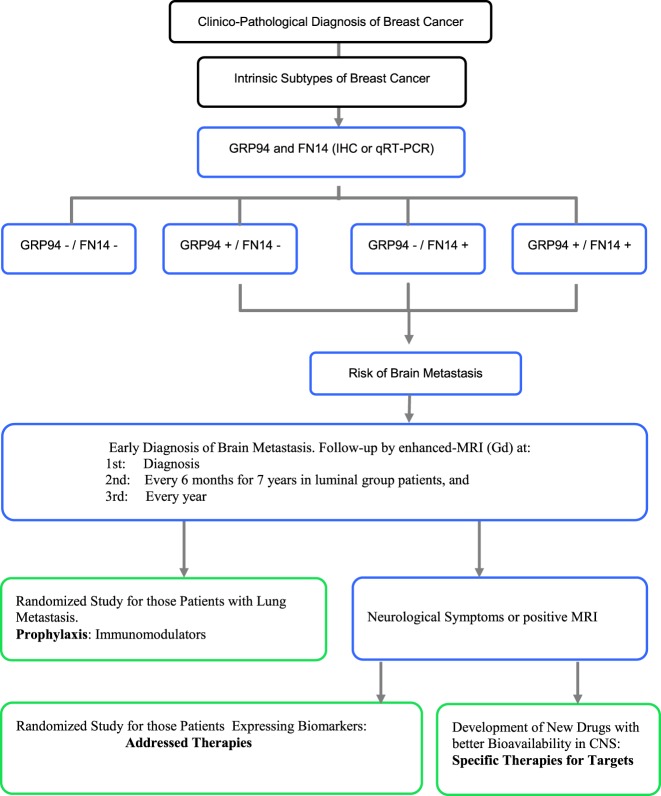
Proposed protocol from diagnosis to prophylaxis and/or treatment approach for patients at risk of brain metastasis. *Black*: current diagnosis protocol. *Blue*: proposed follow-up approach for those patients from the *Luminal* group expressing positive biomarkers. *Green*: prophylaxis and therapies based on biomarker expression.

Many studies have reported risk factors for BrM, including Her2 positivity, ER negativity, high proliferative activity, young age and lymph node involvement (38, 39). FN14 and GRP94 comprise a diagnostic tool capable of predicting BrM independently of these classical clinical and pathological parameters. The long-term BrM-free survival of *Luminal* group patients when biomarkers are negative suggests the usefulness of including both biomarkers to stratify patients that might benefit from magnetic resonance imaging (MRI-Gd) follow-up. This should be considered for at least 7 years (about 80 months) after diagnosis, the period after which BrM relapse in the *Luminal* group stabilizes (Figure 2). Therefore, we suggest that intrinsic subtypes of breast cancer plus FN14 and GRP94 expression can provide a reliable assessment of BrM risk, facilitating early diagnosis through follow-up of the patient’s evolution (Figure 3). Even patients from the non-*Luminal* group could benefit from stratification using FN14 and GRP94 biomarkers. This is not surprising because in combination, they are good predictors of BrM progression in *TN* breast carcinomas (27). Consequently, if these findings were confirmed in further studies, it would also enable us to apply a specific clinical and therapeutical algorithm to improve breast cancer patients’ follow-up.

Although the armamentarium available for BrM treatment is limited, there are reasons to be optimistic because emerging therapies have shown promise in preclinical and early clinical settings (40–42). Moreover, a protocol that included an MRI procedure might provide indications for early surgery and/or radiosurgery when BrM is small to minimal and/or for the design of a new approach in prophylactic systemic protocols (e.g., to replace or add another drug and/or biological compound that crosses the blood–brain barrier to avoid the growth of a clinical BrM) as well as for the design of a new protocol as a prophylactic approach.

Patients with Her2 positivity in primary tumor are usually treated with trastuzumab after delivering chemotherapy, obtaining a better systemic response (43). In our series, 99% of patients did not receive trastuzumab as adjuvant therapy. Thus, an interesting approach would be to study the relationship between FN14 and GRP94 expression and BMFS in those patients belonging to the *Lum/Her2*+ and *Her2-enriched* subtypes who have received trastuzumab. In our multicenter series, GRP94 and FN14 expression might improve breast cancer survival by predicting BrM. In particular, FN14 has a similar sensitivity and specificity to that of Her2.

Immunophenotypic changes associated with antitumor activity have been observed with anti-TWEAK antibody treatment in mice and a phase I multicenter trial of RG7212 monotherapy in patients with FN14-expressing advanced solid tumors has been initiated, with good tolerability and favorable pharmacokinetics (44). Therefore, these molecules might be good candidates to develop new drugs to treat or prevent BrM according to the tumor-associated risk of breast cancer patients.

In itself, BrM is an exclusion criterion for most prospective trials, limiting the possibility of developing new therapies (45). Moreover, therapies are usually started when symptoms appear, limiting treatment options and success (41). We propose a new classification that provides a standard approach for clinical decision-making about CNS metastases at early diagnosis when adjuvant chemotherapy and radiosurgery are more effective (46). Furthermore, evaluation of new and more specific biomarkers in primary tumor may be a promising field of research due to the high impact that these might have in the future as regards facilitating the design of new therapeutic strategies to either prevent or treat this life-threatening event.

## Ethics Statement

This study was approved by Comité etico de Investigación Clínica del Hospital Clínico de Barcelona.

## Author Contributions

AM-A designed the study hypothesis according to both ER status and biomarkers expression, collecting all available clinical and pathological information from patients’ reports and designing the several clinicopathological variables in a new wider and more complete database. VH participated in the follow-up of tissue microarrays and collecting and organizing the whole information emerged from biomarkers expression. FM and RB, as radiation oncologists, and AU, MG-G, and MS, as medical oncologists, contributed with a wide registered clinical follow-up of patients and they offered important clinical data from clinical reports. NB, NV, XA, and EC, as pathologists, contributed with the tissue microarrays analysis and categorizing the level of expression of biomarkers in all studied patients. DC carried out all statistic analysis. AS is the leader and responsible of the project, leading, coordinating, and checking the different steps of the study and assisted in writing the manuscript.

## Conflict of Interest Statement

The authors declare that the research was conducted in the absence of any commercial or financial relationships that could be construed as a potential conflict of interest.

## Abbreviations

BrM, brain metastasis; ER, estrogen receptor; PR, progesterone receptor; TN, triple negative; BCBrM, breast cancer brain metastasis; BrMFS, brain metastasis-free survival; NBrM, non-brain distant metastases; WoM, without metastases; NBrWoM, non-brain distant metastases and without metastases; HG, histological grade.
