# Supplementary material for: Predictive and Prognostic Brain Metastases Assessment in Luminal Breast Cancer Patients: FN14 and GRP94 from Diagnosis to Prophylaxis
Source: Front Oncol. 2017 Dec 1;7:283. doi: 10.3389/fonc.2017.00283 (PMC5716976; doi:10.3389/fonc.2017.00283)
Supplement: Supplementary file 1 [file data_sheet_1.doc]

**SUPPLEMENTARY DATA**

**Patients**

We analyzed the distribution of all our patient series (n=318) according to the presence of metastases and the known risk factors, stratifying the patients in three different groups of progression patterns: patients with *brain metastases* (BrM), with or without metastases at other sites; patients with *non-brain distant metastases* (NBrM), patients with metastasis in bones and/or liver and/or lungs and/or non-regional lymph nodes, but not in brain; and patients *without metastases* (WoM). The main distinctive parameters that characterized the BrM group were *age*, whereby below 50 years old was significantly different (p=*0.001*); *hormone receptor negativity* (both ER [p<*0.0001*] and PR [p<*0.0001*]), an attribute of tumors that developed BrM in contrast to tumors from NBrM and WoM patients; and *Her2 positivity* and a *high Ki-67 index* (p=*0.01* and p<*0.0001*, respectively). Other parameters, such as *tumor size* (p=*0.001*), *histological grade* (p<*0.0001*) and *lymph node involvement* (p<*0.0001*) were similar among BrM and NBrM patients, but different in WoM patients (Supplementary Table S1).

From these 318 patients of our serie, 107 patients were after excluded because of the lack of some data to define the molecular subtypes. Therefore, we only took into account 211 for the subclassification analysis (Supplementary Table S2 and S3).

Patient age at diagnosis ranged between 26 and 86 years old (mean: 55 years old). By age group, 89.6% of patients were ≥ 40 years old, and 10.4% were less than 40. Follow-up ranged from 9 to 146 months (mean: 74.3 months). Only 2.8% of patients had distant metastasis at diagnosis.

The chemotherapeutic protocols were stratified as “taxanes” when paclitaxel or docetaxel were included in the protocol, whereas therapies with others chemotherapeutics were stratified as “non-taxanes”.

25.7% (54/210) of patients received neoadjuvant chemotherapy. We used protocols with anthracyclines (doxorubicin or epirubicin) (n=38), taxanes (n=1), anthracyclines plus taxanes (n=12), anthracyclines plus taxanes plus gemcitabine (n=1) and CMF (cyclophosphamide + methotrexate + 5’ fluorouracil) (n=2); one case was missing. Surgery was carried out on 209 patients (99.0%), 157 of whom (74.4%) received conservative treatment and 52 (24.6%) a mastectomy; two patients did not undergo surgery. 63.2% (129/204) of patients received adjuvant chemotherapy: protocols with CMF (n=36), anthracyclines (n=22), anthracyclines plus CMF (n=47), taxanes (n=14) and anthracyclines plus taxanes (n=10); seven cases were missing. Adjuvant hormonal therapy was prescribed in 60.6% (126/208) of patients: tamoxifen (n=107) and other hormonal schedules (n=19) (patients treated with anastrozole, letrozole, exemestane, toremifene, tamoxifen plus exemestane or letrozole plus LHRH analog); three cases were missing.

99% of patients (207/209) did not receive trastuzumab as adjuvant therapy; two patients were missing. Postoperative radiotherapy was delivered in 180/209 patients (86.1%) and not indicated in 29 patients (13.9%). Treatment was delivered in whole breast after conservative surgery (overall dose of 50 – 50.4 Gy with standard fractionation, 1.8 – 2 Gy per fraction) or in chest wall after mastectomy with the same schedule when indicated. Tumor bed boost in breast or chest wall with an additional dose of 10 – 20 Gy was delivered (2 Gy per fraction), as well as irradiation of regional lymph nodes (overall dose of 50 – 50.4 Gy with standard fractionation, 1.8 – 2 Gy per fraction), when indicated.

11.8% of patients (25/211) suffered local relapse, whereas only 6.2% (13/211) had regional nodal relapse. Distant metastatic relapse occurred in 42.2% (89/211) of patients; of these, 52 patients (58.4%) developed brain metastasis, 32 (36.0%) lung metastasis, 37 (41.6%) liver metastasis, 27 (30.3%) non-regional lymph node metastasis and 57 (64.0%) bone metastasis; 22 patients (24.7%) presented metastasis at other sites. 57.8% (122/211) of patients had no metastatic progression after a minimum follow-up of five years (Supplementary Table S2).

According to the pathological characteristics (see Supplementary Table S3), tumor size at diagnosis was ≤ 20 mm in diameter in more than 50% of patients (121/201); in ten patients, tumor size was not reported. The histological grade (HG), n=201, was mainly grade 2 (49.2%) or 3 (47.8%); ten patients without reported data. According to histological type, the most common phenotype was infiltrating ductal carcinoma in 96.7% (204/211) of patients, followed by lobular in 2.4% (5/211) and other (mucinous or medullary) in 0.9%. Axillary lymphatic nodes (ALN) were evaluated in 204 patients: 54.9% had no regional lymphatic involvement (pN0), 24.0% had 1 to 3 positive axillary nodes and 21.1% had ≥ 4 axillary nodes involved.

The involvement of lymph nodes was missing in seven patients. ER were positive in 67.8% (143/211) of patients, and progesterone receptors (PR) were positive in 60.3% (126/209) of patients, with two patients missing.

The Ki-67index was analyzed in 169 patients, 53.2% of whom scored < 14% and 46.8% scored ≥ 14%; these data were missing in 42tumors. Her2 was positive in 19.0% of analyzed tumors (40/211). Patients distribution according to molecular subtype is exposed as follows: 40.3% luminal A, 18.5% luminal B, 9.0% luminal/Her2+, 10.0% Her2-enriched and 22.2% triple negative.

**Supplementary Tables**

**Supplementary Table S1. Distribution of patients with brain metastasis (BrM), other metastases (NBrM) and non-metastasis (WoM) according to clinical and pathological parameters of the primary breast tumor.**

^1^Patients included in these three categories (N=313/318).

^2^Patients included in the “non-brain distant metastases” category were those with metastasis in one or more of the following organs: bone, lung, liver and non-regional lymph nodes, but without brain metastasis. In this category, five patients (5/318) were previously excluded because they only had skin metastasis (n=2), pleural metastasis (n=2) or meningeal metastasis (n=1). Each category had a variable number of missing patients.

**Supplementary Table S2. Clinical characteristics of breast cancer patients.**

^1^Pleura, skin, meninges, soft tissue, suprarenal gland, bone marrow, peritoneum, pericardium, ovary, choroids, spinal cord and/or cervix.

**Supplementary Table S3. Pathological characteristics and molecular subtypes**

**of breast cancer patients.**

^1^Histological type was obtained by means of surgical specimen analysis and/or biopsy.

^2^Mucinous or medullar.

**Supplementary Table S4. Characteristics in patients with breast cancer according to *Luminal* vs *Non-Luminal* groups.**

^1^This group includes all patients with *Luminal A*, *Luminal B* and *Luminal/Her2+* subtypes.

^2^This group includes all patients with *TN* and *Her2-enriched* subtypes. Frequencies of categorical variables were compared among groups using the Chi-square test or Fisher’s exact test where appropriate.

**Supplementary Table S5. Distribution of biomarkers in Luminal and non-Luminal groups according to Her2 status in patients with brain metastases.**

^1^Of 39 patients belonging to this group, one was excluded (biomarker status unknown).

^2^Six patients belonging to Luminal/Her2+ subtype.

^3^Eleven patients belonging to Her2-enriched subtype.

| **Supplementary Table S1** | | | | | | | | | | | |
| --- | --- | --- | --- | --- | --- | --- | --- | --- | --- | --- | --- |
| **Patients (N=313)**^1^ | | | | | | | | | | | |
|  |  | **Brain**  **Metastases** | |  | **Non-Brain**  **Distant Metastases**^2^ | |  | **Without**  **Metastases** | |  | **p-value**  (Chisq^2^-test) |
|  |  | **N** | **(%)** |  | **N** | **(%)** |  | **N** | **(%)** |  |
| **Characterisics** |  | **84** | **(26.8)** |  | **49** | **(15.7)** |  | **180** | **(57.5)** |  |
| **Age (years)** |  |  | |  |  | |  |  | |  |  |
| < 40 |  | 13 | ( 15.5 ) |  | 10 | ( 20.4 ) |  | 11 | ( 6.1 ) |  |  |
| 40 - 49 |  | 29 | ( 34.5 ) |  | 9 | ( 18.4 ) |  | 40 | ( 22.2 ) |  |  |
| ≥ 50 |  | 42 | ( 50.0 ) |  | 30 | ( 61.2 ) |  | 129 | ( 71.7 ) |  | *0.001* |
| *missing* |  | *0* |  |  | *0* |  |  | *0* |  |  |  |
| **Tumor Size (mm)** |  |  | |  |  | |  |  | |  |  |
| ≤ 20 |  | 35 | ( 47.9 ) |  | 19 | ( 41.3 ) |  | 116 | ( 66.7 ) |  |  |
| ≥ 21 |  | 38 | ( 52.1 ) |  | 27 | ( 58.7 ) |  | 58 | ( 33.3 ) |  | *0.001* |
| *missing* |  | *11* |  |  | *3* |  |  | *6* |  |  |  |
| **Histological Grade** |  |  | |  |  | |  |  | |  |  |
| 1 |  | 2 | ( 2.6 ) |  | 1 | ( 2.2 ) |  | 13 | ( 7.7 ) |  |  |
| 2 |  | 20 | ( 26.3 ) |  | 16 | ( 34.8 ) |  | 106 | ( 62.7 ) |  |  |
| 3 |  | 54 | ( 71.1 ) |  | 29 | ( 63.0 ) |  | 50 | ( 29.6 ) |  | *< 0.0001* |
| *missing* |  | *8* |  |  | *3* |  |  | *11* |  |  |  |
| **Axillary Lymph Nodes +** |  |  | |  |  | |  |  | |  |  |
| 0 |  | 25 | ( 31.6 ) |  | 15 | ( 32.0 ) |  | 113 | ( 64.4 ) |  |  |
| 1-3 |  | 24 | ( 30.4 ) |  | 16 | ( 34.0 ) |  | 43 | ( 24.7 ) |  |  |
| ≥ 4 |  | 30 | ( 38.0 ) |  | 16 | ( 34.0 ) |  | 19 | ( 10.9 ) |  | *< 0.0001* |
| *missing* |  | *5* |  |  | *2* |  |  | *5* |  |  |  |
| **Estrogen Receptor** |  |  | |  |  | |  |  | |  |  |
| Negative |  | 45 | ( 56.2 ) |  | 11 | ( 22.9 ) |  | 20 | ( 11.7 ) |  |  |
| Positive |  | 35 | ( 43.8 ) |  | 37 | ( 77.1 ) |  | 151 | ( 88.3 ) |  | *< 0.0001* |
| *missing* |  | *4* |  |  | *1* |  |  | *9* |  |  |  |
| **Progesterone Receptor** |  |  | |  |  | |  |  | |  |  |
| Negative |  | 48 | ( 60.8 ) |  | 16 | ( 34.0 ) |  | 35 | ( 20.8 ) |  |  |
| Positive |  | 31 | ( 39.2 ) |  | 31 | ( 66.0 ) |  | 133 | ( 79.2 ) |  | *< 0.0001* |
| *missing* |  | *5* |  |  | *2* |  |  | *12* |  |  |  |
| **Her 2** |  |  | |  |  | |  |  | |  |  |
| Negative |  | 58 | ( 75.3 ) |  | 41 | ( 87.2 ) |  | 151 | ( 89.9 ) |  |  |
| Positive |  | 19 | ( 24.7 ) |  | 6 | ( 12.8 ) |  | 17 | ( 10.1 ) |  | *0.01* |
| *missing* |  | *7* |  |  | *2* |  |  | *12* |  |  |  |
| **Ki-67 index (%)** |  |  | |  |  | |  |  | |  |  |
| < 14 |  | 3 | ( 11.5 ) |  | 13 | ( 43.3 ) |  | 76 | ( 63.3 ) |  |  |
| ≥ 14 |  | 23 | ( 88.5 ) |  | 17 | ( 56.7 ) |  | 44 | ( 36.7 ) |  | *< 0.0001* |
| *missing* |  | *58* |  |  | *19* |  |  | *60* |  |  |  |

| **Supplementary Table S2** | | | |
| --- | --- | --- | --- |
|  |  | **Overall patients (N =211)** | |
| **Characteristics** |  | **N** | **(%)** |
|  |  |  |  |
| **Year of Diagnosis** |  | 1989 - 2009 | |
| **Age at Diagnosis (years)** |  | Mean: 55 Range: 26 - 86 | |
| **Follow-up (months)** |  | Mean: 74.3 Range: 9 -146 | |
| **Groups of Age (years)** |  |  |  |
| < 40 |  | 22 | (10.4) |
| 40 - 49 |  | 56 | (26.6) |
| ≥50 |  | 133 | (63.0) |
| **Distant Metastasis at Diagnosis** |  |  |  |
| No |  | 205 | (97.2) |
| Yes |  | 6 | (2.8) |
| **Neoadjuvant Chemotherapy** |  |  |  |
| No |  | 156 | (74.3) |
| Taxanes |  | 14 | (6.7) |
| Non-taxanes |  | 40 | (19.0) |
| *missing* |  | *1* |  |
| **Surgery** |  |  |  |
| No |  | 2 | (1.0) |
| Conservative |  | 157 | (74.4) |
| Mastectomy |  | 52 | (24.6) |
| **Adjuvant Chemotherapy** |  |  |  |
| No |  | 75 | (36.8) |
| Taxanes |  | 24 | (11.8) |
| Non-taxanes |  | 105 | (51.4) |
| *missing* |  | *7* |  |

| **Supplementary Table S2 (Continued)** | | | |
| --- | --- | --- | --- |
|  |  | **Overall patients (N =211)** | |
| **Characteristics** |  | **N** | **(%)** |
|  |  |  |  |
| **Adjuvant Hormonotherapy** |  |  |  |
| No |  | 82 | (39.4) |
| Tamoxifen |  | 107 | (51.5) |
| Other |  | 19 | (9.1) |
| *missing* |  | *3* |  |
| **Adjuvant Trastuzumab** |  |  |  |
| No |  | 207 | (99.0) |
| Yes |  | 2 | (1.0) |
| *missing* |  | *2* |  |
| **Postoperative Radiotherapy** |  |  |  |
| No |  | 29 | (13.9) |
| Yes |  | 180 | (86.1) |
| *missing* |  | *2* |  |
| **Local Relapse** |  |  |  |
| No |  | 186 | (88.2) |
| Yes |  | 25 | (11.8) |
| **Regional Relapse** |  |  |  |
| No |  | 198 | (93.8) |
| Yes |  | 13 | (6.2) |
| **Distant Metastasis** |  |  |  |
| No |  | 122 | (57.8) |
| Yes |  | 89 | (42.2) |
| **Distant Metastasis Sites** |  | **89** |  |
| Bone |  | 57 | (64.0) |
| Brain |  | 52 | (58.4) |
| Liver |  | 37 | (41.6) |
| Lung |  | 32 | (36.0) |
| Non-regional Lymph Nodes |  | 27 | (30.3) |
| Other^1^ |  | 22 | (24.7) |

| **Supplementary Table S3** | | | |
| --- | --- | --- | --- |
|  |  | **Overall patients (N =211)** | |
| **Characteristics** |  | **N** | **%** |
|  |  |  |  |
| **Tumor Size (mm)** |  |  |  |
| ≤ 20 |  | 121 | (60.2) |
| ≥ 21 |  | 80 | (39.8) |
| *missing* |  | *10* |  |
| **Histological Grade** |  |  |  |
| 1 |  | 6 | (3.0) |
| 2 |  | 99 | (49.2) |
| 3 |  | 96 | (47.8) |
| *missing* |  | *10* |  |
| **Histological Type^1^** |  |  |  |
| Ductal |  | 204 | (96.7) |
| Lobular |  | 5 | (2.4) |
| Other^2^ |  | 2 | (0.9) |
| **Positive Axillary Nodes** |  |  |  |
| 0 |  | 112 | (54.9) |
| 1 to 3 |  | 49 | (24.0) |
| ≥ 4 |  | 43 | (21.1) |
| *missing* |  | *7* |  |
| **Estrogen Receptors** |  |  |  |
| Negative |  | 68 | (32.2) |
| Positive |  | 143 | (67.8) |
| **Progesterone Receptors** |  |  |  |
| Negative |  | 83 | (39.7) |
| Positive |  | 126 | (60.3) |
| *missing* |  | *2* |  |

| **Supplementary Table S3 (Continued)** | | | |
| --- | --- | --- | --- |
|  |  | **Overall patients (N =211)** | |
| **Characteristics** |  | **N** | **%** |
|  |  |  |  |
| **Ki-67 index (%)** |  |  |  |
| < 14 |  | 90 | (53.2) |
| ≥ 14 |  | 79 | (46.8) |
| *missing* |  | *42* |  |
| **Her 2** |  |  |  |
| Negative |  | 171 | (81.0) |
| Positive |  | 40 | (19.0) |
| **Molecular Subtype** |  |  |  |
| Luminal A |  | 85 | (40.3) |
| Luminal B |  | 39 | (18.5) |
| Luminal / Her 2+ |  | 19 | (9.0) |
| Her 2-enriched |  | 21 | (10.0) |
| Triple negative |  | 47 | (22.2) |

| **Supplementary Table S4.** | | | | | | | | |
| --- | --- | --- | --- | --- | --- | --- | --- | --- |
|  |  | **Patients (N=211)** | | | | |  |  |
|  |  | **Luminal Group^1^ (N=143)** | |  | **Non-Luminal Group^2^ (N=68)** | |  |  |
| **Characteristics** |  | **N** | **(%)** |  | **N** | **(%)** |  | **p- value** |
|  |  |  |  |  |  |  |  |  |
| **Groups of Age (years)** |  | **143** |  |  | **68** |  |  |  |
| < 40 |  | 15 | (10.49) |  | 7 | (10.29) |  |  |
| 40 - 49 |  | 41 | (28.67) |  | 15 | (22.06) |  |  |
| ≥ 50 |  | 87 | (60.84) |  | 46 | (67.65) |  | 0.57 |
| **Tumor Size (mm)** |  | **138** |  |  | **63** |  |  |  |
| ≤ 20 |  | 93 | (67.39) |  | 28 | (44.44) |  |  |
| ≥ 21 |  | 45 | (32.61) |  | 35 | (55.56) |  | *0.002* |
| *missing* |  | 5 |  |  | 5 |  |  |  |
| **Histological Grade** |  | **137** |  |  | **64** |  |  |  |
| 1 |  | 6 | (4.38) |  | 0 | (0.00) |  |  |
| 2 |  | 87 | (63.50) |  | 12 | (18.75) |  |  |
| 3 |  | 44 | (32.12) |  | 52 | (81.25) |  | *<0.0001* |
| *missing* |  | 6 |  |  | 4 |  |  |  |
| **Positive Axillary Nodes** |  | **139** |  |  | **65** |  |  |  |
| 0 |  | 87 | (62.59) |  | 25 | (38.46) |  |  |
| 1 to 3 |  | 32 | (23.02) |  | 17 | (26.15) |  |  |
| ≥ 4 |  | 20 | (14.39) |  | 23 | (35.39) |  | *0.0008* |
| *missing* |  | 4 |  |  | 3 |  |  |  |
| **Neoadjuvant Chemotherapy** |  | **143** |  |  | **67** |  |  |  |
| No |  | 117 | (81.82) |  | 39 | (58.21) |  |  |
| Taxanes |  | 4 | (2.80) |  | 10 | (14.92) |  |  |
| Non-Taxanes |  | 22 | (15.38) |  | 18 | (26.87) |  | *0.0002* |
| *missing* |  | 0 |  |  | 1 |  |  |  |
| **Surgery** |  | **143** |  |  | **68** |  |  |  |
| No |  | 0 | (0.00) |  | 2 | (2.94) |  |  |
| Conservative |  | 119 | (83.22) |  | 38 | (55.88) |  |  |
| Mastectomy |  | 24 | (16.78) |  | 28 | (41.18) |  | *<0.0001* |
| *missing* |  | 0 |  |  | 0 |  |  |  |

| **Supplementary Table S4 (Continued)** | | | | | | | | |
| --- | --- | --- | --- | --- | --- | --- | --- | --- |
|  |  | **Patients (N=211)** | | | | |  |  |
|  |  | **Luminal Group^1^ (N=143)** | |  | **Non-Luminal Group^2^ (N=68)** | |  |  |
| **Characteristics** |  | **N** | **(%)** |  | **N** | **(%)** |  | **p- value** |
|  |  |  |  |  |  |  |  |  |
| **Adjuvant Chemotherapy** |  | **143** |  |  | **61** |  |  |  |
| No |  | 64 | (44.76) |  | 11 | (18.03) |  |  |
| Taxanes |  | 15 | (10.49) |  | 9 | (14.76) |  |  |
| Non-Taxanes |  | 64 | (44.75) |  | 41 | (67.21) |  | *0.001* |
| *missing* |  | 0 |  |  | 7 |  |  |  |
| **Adjuvant Hormonotherapy** |  | **142** |  |  | **66** |  |  |  |
| No |  | 20 | (14.08) |  | 62 | (93.94) |  |  |
| Tamoxifen |  | 103 | (72.54) |  | 4 | (6.06) |  |  |
| Other |  | 19 | (13.38) |  | 0 | (0.00) |  | *<0.0001* |
| *missing* |  | 1 |  |  | 2 |  |  |  |
| **Postoperative Radiotherapy** |  | **143** |  |  | **66** |  |  |  |
| No |  | 18 | (12.59) |  | 11 | (16.67) |  |  |
| Yes |  | 125 | (87.41) |  | 55 | (83.33) |  | 0.43 |
| *missing* |  | 0 |  |  | 2 |  |  |  |
| **Local Relapse** |  | **143** |  |  | **68** |  |  |  |
| No |  | 131 | (91.61) |  | 55 | (80.88) |  |  |
| Yes |  | 12 | (8.39) |  | 13 | (19.12) |  | *0.024* |
| *missing* |  | 0 |  |  | 0 |  |  |  |
| **Regional Relapse** |  | **143** |  |  | **68** |  |  |  |
| No |  | 141 | (98.60) |  | 57 | (83.82) |  |  |
| Yes |  | 2 | (1.40) |  | 11 | (16.18) |  | *<0.0001* |
| *missing* |  | 0 |  |  | 0 |  |  |  |
| **Distant Metastases** |  | **143** |  |  | **68** |  |  |  |
| No |  | 104 | (72.73) |  | 18 | (26.47) |  |  |
| Yes |  | 39 | (27.27) |  | 50 | (73.53) |  | *<0.0001* |
| *missing* |  | 0 |  |  | 0 |  |  |  |

| **Supplementary Table S5.** | | | | | | | | | | | | | | | | |
| --- | --- | --- | --- | --- | --- | --- | --- | --- | --- | --- | --- | --- | --- | --- | --- | --- |
|  |  | **Patients (N=52)** | | | | | | | | | | | | | | |
|  |  | **Luminal Group (N=13)** | | | | | | |  | **Non-Luminal Group (N=39)^1^** | | | | | | |
|  |  | **Her2 +**  (N=6)^2^ | |  | **Her2 –**  (N=7) | |  |  |  | **Her2 +**  (N=11)^3^ | |  | **Her2 –**  (N=27) | |  |  |
|  |  |  |  |  |  |  |
| **Biomarker** |  | **N** | **(%)** |  | **N** | **(%)** |  | p-value (Fisher**)** |  | **N** | **(%)** |  | **N** | **(%)** |  | p-value (Fisher) |
| FN14 + |  | 4 | (66.7) |  | 3 | (42.9) |  | 0.59 |  | 7 | (63.6) |  | 5 | (18.5) |  | *0.017* |
|  |  |  |  |  |
| GRP94 + |  | 5 | (83.3) |  | 5 | (71.4) |  | 1.00 |  | 8 | (72.7) |  | 14 | (51.9) |  | 0.30 |
|  |  |  |  |  |
|  | |  |  |  |  |  |  |  |  |  |  |  |  |  |  |  |

**Supplementary Figures**

**Supplementary Figure S1. Metastasis-free survival according to molecular group (Luminal vs non-Luminal)**

**(A)**. Non-brain metastasis-free survival once molecular subtypes had been grouped into two molecular groups (*Luminal* vs non-*Luminal*). There were significant differences in non-brain metastasis-free survival between the *Luminal* and non-*Luminal* groups, with the latter presenting a higher risk (HR=4.01; 95%CI:2.52-6.38; p<*0.0001*) (Kaplan-Meier method, using the Cox proportional hazards model).

**(B).** Brain metastasis-free survival once the molecular subtypes had been grouped into two molecular groups (*Luminal* vs Non.*Luminal*). Non-*Luminal* group showed a higher risk of brain metastasis with significant differences (HR=10.57; 95%CI:5.60-19.96; p<*0.0001*) (Kaplan-Meier method, using the Cox proportional hazards model).

**Supplementary Figure S2.**  **Metastasis-free survival according to molecular subtypes.**

The results are referenced with regard to the TN subtype.

**(A).** In the NBrM group, the remaining subtypes presented less risk of BrM, but this was only statistically significant in *Luminal* subtypes: *Luminal/Her2+* (HR=0.41; 95%CI:0.18-0.96; p=*0.039*), *Luminal B* (HR=0.33; 95%CI:0.17-0.64; p=*0.0012*) and *Luminal A* (HR=0.16; 95%CI:0.08-0.30; p<*0.0001*) subtypes. In the *Her2-enriched* subtype, differences were not significant (HR=0.81; 95%CI:0.41-1.60; p=0.53).

**(B).** Similarly, in the BrM group, *Luminal* tumors presented a statistically significant lower risk of brain metastasis: *Luminal/Her2+* (HR=0.33; 95%CI:0.13-0.80; p=*0.014*), *Luminal B* (HR=0.13; 95%CI:0.05-0.33; p*<0.0001*) and *Luminal A* (HR=0.02; 95%CI:0.005-0.09;, p*<0.0001*). In contrast, differences in brain metastasis progression in the *Her2-enriched* subtype were not significant (HR=0.79; 95%CI:0.40-1.55; p=*0.49*).

**Supplementary Figure S1**

**(A)**

**(B)**

**Supplementary Figure S2**

**(A)**

**(B)**
